# Dendritic cell therapy for oncology roundtable conference

**DOI:** 10.1186/1476-8518-9-1

**Published:** 2011-01-12

**Authors:** Sandra Tuyaerts

**Affiliations:** 1 Laboratory of Experimental Gynaecology, Department of Woman & Child, Catholic University of Leuven (K.U. Leuven), Belgium

## Abstract

2-3 September 2010, Brussels, Belgium

The Dendritic Cell Therapy for Oncology Roundtable Conference was organized by Reliable Cancer Therapies and moderated by Prof. Dr. Steven De Vleeschouwer. The organizer, Reliable Cancer Therapies, is a Swiss non-profit organization that provides information on evidence-based cancer treatments and funding for the development of a selection of promising cancer therapies. In order to be able to give valuable information about dendritic cell (DC) therapy to patients and physicians, the organizing committee felt it necessary to organize this conference to get an up-to-date status of the academic DC therapy field, collect ideas to guide patients towards clinical trials and to induce cross-fertilization for protocol optimization. In total, 31 experts participated to an in-depth discussion about the status and the future development path for dendritic cell vaccines. The conference started with general presentations about cancer immunotherapy, followed by comprehensive overview presentations about the progress in DC vaccine development achieved by each speaker. At the end of the meeting, a thorough general discussion focused on key questions about what is needed to improve DC vaccines. This report does not cover all presentations, but aims to highlight selected points of interest, particularly relating to possible limitations and potential approaches to improvement of DC therapies specifically, and also immunotherapeutic interventions in general terms.

## General topics of cancer vaccination

The conference started with an introductory lecture by Chris Schmidt, addressing key aspects why cancer vaccines do not work as expected. Initially, cancer vaccines were tested in the systemic disease setting and after obtaining positive results in this patient population, moved to the minimal residual disease (MRD) setting because of the hypothesis that if a vaccine worked in a macroscopic disease setting, it should work better in the MRD setting (less immunosuppression). However, in the MRD setting adjuvant vaccines have often failed, which may be due to too short follow-up periods. On the other hand, it could also be postulated that the systemic disease setting just indicates the availability of large amounts of antigen, where the vaccine can trigger an anti-vaccine T cell response that attack the tumor and in this way activate a second wave of anti-tumor CTL. This relates to the vaccine targets: a meta-analysis of all immunotherapy trials indicated that the rate of objective clinical responses is higher when undefined antigens are used as compared to defined antigens [1,2]. This argues that many targets need to be attacked but that till now: (1) we do not know which are the relevant targets; or (2) other molecules are present in tumor extracts that influence the regulatory environment in a way that defined antigens cannot; or (3) effective immune responses only act at sites of macroscopic tumor. Another reason why cancer treatments in general and vaccines in specific fail to cure patients could be related to the issue of timing of therapy. This was addressed by Brendon Coventry, who presented data indicating that the endogenous anti-tumor immune response follows a cyclical pattern (measured by CRP) which is dependent on antigen persistence and that this cycle could likely potentially correlate with numbers of effector T cells and regulatory T cells (Treg) over time. Preliminary evidence suggests that efficacy of chemo-, radio- or immunotherapy could be boosted by appropriate timing to putative 'therapeutic windows' in the individual patient's CRP cycle [3]. Furthermore, cancer vaccines do not encounter a naïve environment, but instead need to counter tumor-induced tolerogenic mechanisms. Treg are major players in immunosuppression, but in humans there are controversies as to the exact phenotype of these cells. Increased percentages of Treg have been described in various malignancies, but it is also important to take into account that absolute lymphocyte counts and percentage of CD4^+ ^T cells are also abnormal in cancer patients. Therefore, it is necessary to measure absolute numbers of Treg in blood instead of percentages. To date however, no regimen is available to reproducibly deplete Treg before proceeding to cancer vaccination. This could be due to the fact that we are looking at total Treg, which may not all be functional? This urges us to focus on identifying functional Treg. To this regard, Michael Quinn presented results about TNFRII^+ ^Treg, which express higher levels of FoxP3 than conventional Treg, express CCR4 (migration to tumor) and CCR7 (migration to lymph nodes -interferes with priming of anti-tumor responses). Furthermore, preliminary results indicated that although total Treg levels increase during chemotherapy, TNFRII^+ ^Treg are selectively depleted by chemotherapy and the remaining TNFRII^+ ^Treg express lower levels of FoxP3, that have a reduced suppressive capacity.

## Feasibility and safety of DC vaccination in cancer patients

Most clinical trials conducted to date with DC vaccines are phase I feasibility studies. Despite many alterations in the immune system of cancer patients, all presenters reported that it was feasible to generate the desired numbers of DC to complete the planned vaccination scheme in most patients. However, Allan Dietz pointed out that we need to cautiously report the characteristics of the generated DC, because the phenotype of DC in trials is consistently different than in preclinical studies with normal donors. This deficiency in DC differentiation in tumor bearing patients is independent of tumor type and maturation method and there appear to be tumor-specific conditions for optimal maturation of DC.

In most of the presented studies, DC vaccination was safe, well tolerated, with minimal side effects. However, Dagmar Marx and Stefaan Van Gool reported the occurrence of some grade III/IV adverse events in a minority of patients, but this could possibly be related to disease localization in the brain (both studies in brain tumors) [4,5]. Some participants reported the induction of auto-immune effects like vitiligo, but in general most participants agreed that some degree of auto-immunity is probably beneficial for DC vaccine efficacy.

## Patient selection

As outlined above, it was originally hypothesized that DC vaccines should perform better in the MRD setting as compared to patients with widespread disease. However, this does not always appear to be the case, which is possibly due to the altered immune system in cancer patients and the high degree of immunosuppression. Therefore, it would be ideal if we could select patients that are likely to benefit from DC vaccination. To this regard, Angus Dalgleish reported on a study where pre-vaccine sera from responders and non-responders were compared. This led to the identification of molecules that can distinguish responder patients from the non-responder group with 67% sensitivity and 100% specificity. All identified molecules are pro-inflammatory and are increased in the non-responder population; however, no details were given about the exact nature of these molecules. Bart Neyns reported that baseline CRP, LDH, and WHO performance status can also identify melanoma patients likely to benefit from DC vaccinations. Chris Schmidt showed that low S-100B predicts response to treatment in melanoma. Allan Dietz presented data showing that suppressive monocytes are increased in several malignancies and these cells mediate a global immune paralysis. Suppressive monocytes were found to be prognostic for cancer survival independent of therapy [6,7]. Selection of patients on the basis of the CRP inflammatory cycle was also raised by Brendon Coventry as a possibility for patient selection with respect to timing of administration and targeting of therapy, in order to induce the desired immune response.

## Clinical effects of DC vaccines

Clinical responses are of course largely affected by the setting in which vaccination occurs (measurable disease versus MRD), the type of cancer and the life expectancy of the patients. Furthermore, the evaluation of clinical efficacy can be impeded by the application of concurrent or subsequent therapeutic regimens. Moreover, most trials conducted to date are phase I clinical trials in which clinical efficacy is very difficult to assess. Nevertheless some promising results have been obtained. In general, long-term objective responses (CR or PR) are observed in a minority of patients, while a greater proportion of patients presents with disease stabilization. In particular, Bart Neyns pointed out that stabilization is often followed by disease regression and that clinical responses could be delayed even up to several months after initiation of treatment, indicating an immune-related response pattern (as described for anti-CTLA4 therapy). Therefore, immune-related response criteria (irRC) may be more relevant than RECIST/WHO criteria for the assessment of anti-tumor activity [8,9]. Furthermore, most participants agree that long-term disease stabilization resulting in prolonged survival is also a relevant clinical outcome, which is of benefit for patients. The typical slow response pattern observed also indicates that DC vaccination is no option for patients with rapidly progressing disease and that patient selection is critical.

## Immune response monitoring after immunotherapy

The rationale behind DC-based immunotherapy is that injected DC will induce a tumor-specific immune response resulting in tumor shrinkage/clearance. So, ideally we should be able to identify patients that respond to therapy by analyzing the anti-tumor immune response generated by the DC vaccine. However, to date, limited studies show a correlation between immune responders and clinical responders, indicating that either we are not analyzing the right portion of the immune response or that the mode of action (MOA) of DC vaccines is not as expected. Chris Schmidt emphasized that we should monitor responses to the tumor and not only to the vaccine and they also observed that DC from complete responders surprisingly had lower IL-12p70/IL-10 ratios, which is not conform the stereotype of immunogenic DC. Viggo Van Tendeloo and Massimo Di Nicola reported that higher levels of activated NK cells correlated with clinical response, indicating that DC vaccines not only act on the T cell response but also on innate immunity [10,11]. Massimo Di Nicola used killed autologous tumor cells to load DC and observed that killed tumor cell preparations from responding patients showed higher calreticulin and heat shock protein 90 expression compared to non-responders, indicating immunogenic tumor cell death is needed to obtain responses with tumor lysate pulsed DC [12]. The group of Jolanda de Vries developed a skin test where the skin-infiltrating lymphocytes (SKILS) in DTH sites are tested for antigen specificity. The presence of tumor-specific SKILS correlated with survival and can thus be used as a predictor of clinical response [13,14]. Despite the fact that in most studies a correlation between immune response and clinical efficacy could not be established, Antoni Ribas suggested that, in future trials, immune monitoring studies need to be expanded and associated with the observed clinical responses to assess the underlying immune mechanisms and the MOA of DC vaccines.

## Maximizing DC biology potential

Initial studies on DC vaccination predominantly used immature or cytokine matured DC. However, since then several improvements have been developed to enhance the DC's capacity to stimulate T cells and even circumvent immunosuppression. Bart Neyns presented data from so-called TriMix DC, i.e. DC transfected with mRNA encoding constitutively active TLR4 (caTLR4), CD40L and CD70. These TriMix DC are highly mature and secrete high levels of IL-12p70. When co-transfected with TAA encoding mRNA, TriMix DC induce potent TAA-specific CTL in advanced melanoma patients [15]. CD4^+ ^T cells are essential for the induction of potent CTL response, but data concerning Th cell induction by DC vaccines are scarce. Martin Cannon highlighted that we should try to redirect DC-activated Treg responses to anti-tumor Th17 responses since it was shown in ovarian cancer that these 2 cell types are inversely correlated and that tumor-associated polyfunctional Th17 cells are associated with improved clinical outcome [16]. Therefore, they treated DC with IL-15 and a p38 MAPK inhibitor and these cells are capable of inducing TNFα^+ ^FoxP3^- ^IL-17 secreting CD4^+ ^T cells that show reduced PD-1 expression and tumor-specific CTL. DC treated with IL-15 and p38 MAPK inhibitor show decreased expression of B7-H1 and reduced IDO activity. Scott Pruitt presented data of local delivery of immune modulators by DC. Therefore, DC were transfected with RNA encoding the GITRL fusion protein and/or anti-CTLA4 mAb. These mediators are then produced locally by DC, thereby preventing side effects that occur with systemic administration as observed with anti-CTLA4 mAb [17]. Previous studies have shown that injected DC poorly traffic to lymph nodes where they should interact with T cells. Jeffrey Weber therefore designed a system to attract T cells to the injection site and induce in this way and "artificial lymph nodal aggregate" for T cell priming. To achieve this, DC are adenovirally transduced with CCL21/SLC and pulsed with peptides. The trial is still ongoing, but immunohistochemical analysis of the injection site shows substantial T cell infiltration. Another intriguing approach consists of redirecting the immune response from an anti-tumor response to an anti-viral response. Dagmar Marx and Volker Schirrmacher reported studies combining DC with the oncolytic Newcastle Disease Virus (NDV). NDV mediates lysis of tumor cells, either *in vitro *to pulse DC with viral oncolysates or *in vivo *to provide a supply of tumor antigens in the body of the patients. The tumor cells then present viral antigens to which a more potent immune response can be generated since there is less tolerance and the virus itself also drives DC polarization towards Th1 inducers [18-21].

## Combination strategies

Increasing evidence suggests that DC vaccines on their own are not capable to induce tumor regression in a substantial amount of patients, but should instead be used in combinatorial approaches. Many potential rationales exist for combination of DC with chemotherapy. Chemotherapy could have effects on MDSC and/or Treg, could increase the susceptibility of tumor cell apoptosis or lead to increased tumor cell immunogenicity, as shown by Angus Dalgleish. He also postulates that combination with immune response modifiers like low dose IL-2, Imiquimod or IMiDs would also be promising. Antoni Ribas showed data from a trial combining DC vaccines with the anti-CTLA4 mAb Tremelimumab, in which 4/16 patients experienced long term responses [22]. Jolanda de Vries presented data about the combination of DC vaccines with Daclizumab pre-treatment to deplete Treg, but although TAA-specific T cells could be generated, these T cells had impaired effector functions and the combination did not result in a significant effect on survival [23]. An attractive idea is to combine DC vaccines with adoptive T cell transfer, where DC vaccines could first prime tumor-specific T cells *in vivo*, which could subsequently be expanded *ex vivo *and then be given back to the patients. This type of combination was presented by Isabel Poschke and Gunnar Kvalheim, but studies are still ongoing. This type of combination is also complicated by the high costs. Another rationale is to incorporate DC vaccination in the standard of care (SOC) treatment if available. Surasak Phuphanich and Stefaan Van Gool presented results from this approach, with positive effects on patient survival [4,5,24-26].

## Approved immunotherapeutic vaccines

Provenge^® ^or Sipuleucel-T from Dendreon received the first approval for a cell-based immunotherapy and hence is an important step in the development of similar strategies. This therapeutic vaccine was briefly discussed during the meeting. Historically, this vaccine is categorized as a DC vaccine, although it does not consist of "pure" DC; hence the term antigen pulsed activated peripheral blood mononuclear cells is probably more correct. The latest phase III trial demonstrated that the effect is merely noted on overall survival (4 month survival benefit), with less evidence of an antitumor effect (1/341 PR, 3% of patients with 50% PSA decrease). Two thirds of patients receiving sipuleucel-T developed antibody responses, and nearly three fourths had T cell proliferative responses. Survival was improved for patients who had an antibody response but not for those with a T cell response. Although these data are encouraging, they also highlight again that still very little is known about the real MOA of such immunotherapeutics. Furthermore, the high cost of such treatments may impact its use and further development [27,28]. It would also be very interesting to investigate whether the effect of Provenge^® ^can be enhanced by combination with approaches aiming at modulating the immunosuppressive environment.

## Discussion

The general discussion of the meeting focused on a few key questions to rapidly move DC vaccination forward.

The first part of the discussion concentrated on the discrepancy between proof of principle and proof of efficacy. Most participants agree that the desired endpoint is clinical activity (OS, PFS, overall response rate) and that immune monitoring is less important. The main problem with this point of view is that efficacy data are difficult to obtain in phase I clinical trials, so a controlled trial would need to be performed. Other variables that can affect the selection of the desired endpoint is whether the patient population is homogeneous or heterogeneous, whether there is bulk tumor or minimal residual disease... It is probably best to carefully select patients and assess safety and MOA in a phase I trial and then rapidly move to randomized controlled trials. Alternatively, vaccination could be added to the SOC and comparative effectiveness research could be performed. When focusing on the response rate, responses (CR, PR or even SD) should always be prolonged in time. Besides assessing clinical efficacy, immune monitoring studies are critical to understand the MOA of DC vaccines and also reporting of vaccine characteristics remains crucial in this regard.

Another discussion point focused on the question why clinical data are disappointing. Probably this relates to our ignorance about the MOA of DC vaccines. Till now, there are no convincing data about the timing of vaccination, how frequently we need to vaccinate and for which period of time. Nevertheless, the approval by the FDA of Provenge^®^, the first cellular immunotherapeutic, has paved the way for further development of DC vaccines and will certainly boost the field further.

Furthermore, we urgently need to get more knowledge about how to efficiently skew immune responses towards the desired phenotype for tumor eradication. Studies to resolve these issues are thus warranted. Furthermore, the concept that DC vaccines should be regarded as bystander therapeutics urges us to go ahead with the rational design of combinatorial approaches with chemotherapeutic regimens, other immunotherapeutic regimens aiming at breaking tolerance, immune response modifiers or targeted therapies (anti-angiogenic molecules, STAT3 inhibitors,...).

Next, the discussion moved to the issue of patient selection for inclusion in DC trials. The rationale argues for inclusion of less advanced patients in which we should then be able to follow a tumor marker to assess efficacy. However, is a patient that can mount an immune response really an end stage patient? Furthermore, it is not ethical to treat patients with DC vaccines if they can still benefit from a SOC treatment. Therefore, it was proposed to try to integrate DC vaccination in the SOC treatment if available or try to combine DC vaccination with available treatment approaches. On the other hand, patients should be carefully selected before inclusion in DC trials to obtain maximal benefit as possible, based on baseline characteristics that are known to correlate with improved outcome and on the aggressiveness of the disease progression.

Finally, how can we improve the potential of DC therapy? Ideally, we should move from *ex vivo *generated DC to purified myeloid or plasmacytoid DC or even *in vivo *DC targeting, but before this can be pursued we need to better understand the immunoregulatory network. The use of multiple defined antigens or the whole tumor antigenic spectrum is encouraged to avoid escape and to induce a broad response, even if this implies the risk to induce some auto-immune effects, which seems to be beneficial for DC vaccine efficacy. Furthermore, studies should focus on the induction of broad polyfunctional immune responses encompassing both innate and adaptive immunity.

In conclusion, this meeting brought together experts in different aspects of DC vaccine development and provided a platform for exchange of ideas, interesting new findings, encountered barriers and potential innovative new approaches. Hopefully this cross-fertilization between scientists will result in the translation to successful clinical trials that can move forward the DC vaccination approach.

## List of abbreviations

CR: complete response; CRP: C-Reactive Protein; CTL: cytotoxic T lymphocyte; DC: dendritic cell; DTH: delayed type hypersensitivity; IMiD: immunomodulatory drug; irRC: immune-related response criteria; LDH: lactate dehydrogenase; MDSC: myeloid-derived suppressor cells; MOA: mode of action; MRD: minimal residual disease; NDV: Newcastle disease virus; NK: natural killer cell; OS: overall survival; PFS: progression-free survival; PR: partial response; RECIST: response evaluation criteria in solid tumors; SD: stable disease; SKILs: skin-infiltrating lymphocytes; SOC: standard of care; TAA: tumor-associated antigen; Th cell: T helper cell; TLR: toll-like receptor; Treg: regulatory T cell; WHO: World Health Organization.

## Competing interests

The author has no relevant affiliations or financial involvement with any organization or entity with a financial interest in or financial conflict with the subject matter or materials discussed in the manuscript. This includes employment, consultancies, honoraria, stock ownership or options, expert testimony, grants or patents received or pending, or royalties.

No writing assistance was utilized in the production of this manuscript.

## Appendix: Meeting participants

Zwi Berneman, University Hospital, Antwerp, Belgium

Martin Cannon, University of Arkansas for Medical Sciences, Little Rock, USA

Raja Choudhury, Karolinska Institute, Stockholm, Sweden

Brendon Coventry, University of Adelaide, Adelaide, Australia

Angus Dalgleish, St George's University of London, London, UK

Jolanda de Vries, Nijmegen Centre for Molecular Life Sciences, Nijmegen, The Netherlands

Allan Dietz, Mayo Clinic, Rochester, USA

Massimo Di Nicola, Fondazione IRCCS Istituto Nazionale Tumori, Milan, Italy

Steve Emery, German Cancer Research Center, Tumor Immunology Program, Heidelberg, Germany

Rolf Kiessling, Karolinska Institute, Stockholm, Sweden

Gunnar Kvalheim, Oslo University Hospital, Oslo, Norway

Dirk Lorenzen, Institute for Tumor Immunology, Duderstadt, Germany

Dagmar Marx, Institute for Tumor Immunology, Duderstadt, Germany

Bart Neyns, Brussels University Hospital, Brussels, Belgium

Surasak Phuphanich, Cedars-Sinai Medical Center, Los Angeles, USA

Isabel Poschke, Karolinska Institute, Stockholm, Sweden

Scott K. Pruitt, Duke University Medical Center, Durham, USA

Michael A. Quinn, University of Melbourne, Melbourne, Australia

Antoni Ribas, University of California at Los Angeles, Los Angeles, USA

Volker Schirrmacher, German Cancer Research Center, Tumor Immunology Program, Heidelberg, Germany

Chris Schmidt, Queensland Institute of Medical Research, Brisbane, Australia

Inge Marie Svane, Department of Oncology, Copenhagen University Hospital, Denmark

Kris Thielemans, Brussels University Hospital, Brussels, Belgium

Stefaan Van Gool, Pediatric Neuro-oncology, University Hospital Leuven, Catholic University of Leuven, Belgium

Viggo Van Tendeloo, University Hospital, Antwerp, Belgium

Jeffrey S. Weber, H. Lee Moffitt Cancer Center, Tampa, USA
